# Integrating tissue proteomics and bioactive lipid analysis of ovine tissue samples identifies functional relations of bioactive lipids

**DOI:** 10.3389/fphar.2026.1959280

**Published:** 2026-10-29

**Authors:** Gerhard Hagn, Florien Jenner, Andrea Bileck, Sinan Gültekin, Mats Hamberg, Thomas Mohr, Christopher Gerner, Iris Gerner

**Affiliations:** 1 Institute of Analytical Chemistry, Faculty of Chemistry, University of Vienna, Vienna, Austria; 2 Vienna Doctoral School in Chemistry (DoSChem), University of Vienna, Vienna, Austria; 3 Equine Surgery Unit, Department of Companion Animals and Horses, University of Veterinary Medicine Vienna, Vienna, Austria; 4 Joint Metabolome Facility, University of Vienna and Medical University of Vienna, Vienna, Austria; 5 Unit of Integrative Metabolomics, Institute of Environmental Medicine, Karolinska Institutet, Stockholm, Sweden; 6 Larodan Research Laboratory, Karolinska Institutet, Stockholm, Sweden

**Keywords:** 4-HETE, inflammation resolution, lipid functions, mass spectrometry, ovine tissue, oxylipins, polyunsaturated fatty acids (PUFAs)

## Abstract

Bioactive lipids are potent effector molecules that may help to reveal specific information about biological processes. However, analytical challenges and incomplete functional annotations remain as obstacles. We hypothesized that the functional annotation of these lipids may be improved by projecting enriched Gene Ontology (GO) terms derived from proteomics to correlating bioactive lipids. To that aim, thirteen organs and tissues from sheep (*Ovis aries*) were analyzed using mass spectrometry-based untargeted proteomics and bioactive lipid analysis. In total, 4717 proteins and 168 bioactive lipids, including fatty acids and their conjugates, oxylipins (octadecanoids, eicosanoids, and docosanoids), lyso-phosphatidylcholines (LPCs) and lyso-phosphatidylethanolamines (LPEs), endocannabinoids and related fatty amides, and bile acids and related sterol lipids were determined. Thereof, the correct identification of 114 lipids was confirmed using commercially available analytical standards, whereas the identity of the previously uncharacterized oxylipin 4-hydroxy-eicosatetraenoic acid (4-HETE) was verified through in-house chemical synthesis. Co-expression and clustering analyses are consistent with our hypothesis *via* independently corroborating known bioactive lipid functions and suggested novel functional relations. Amongst other observations, 18-HETE and 19-HETE were related to mitochondrial fatty acid beta-oxidation, 11,12-dihydroxy-eicosatetraenoic acid (DiHETE), 15-deoxy-prostaglandin J2 and 5(6)-epoxy-eicosatrienoic acid (EET) were related to chemical synaptic transmission, arachidonoyl ethanolamide phosphate was related to muscle contraction and numerous LPCs and LPEs were related to exosome formation. Tissue levels of polyunsaturated fatty acids (PUFAs), pro-resolving mediators and the newly characterized 4-HETE were related to the cellular protein synthesis and folding machinery. The latter finding suggested that PUFAs and their derivatives support protein synthesis fidelity in addition to efferocytosis, both essential contributors for the effective resolution of inflammation.

## Graphical Abstract

**Figure:**
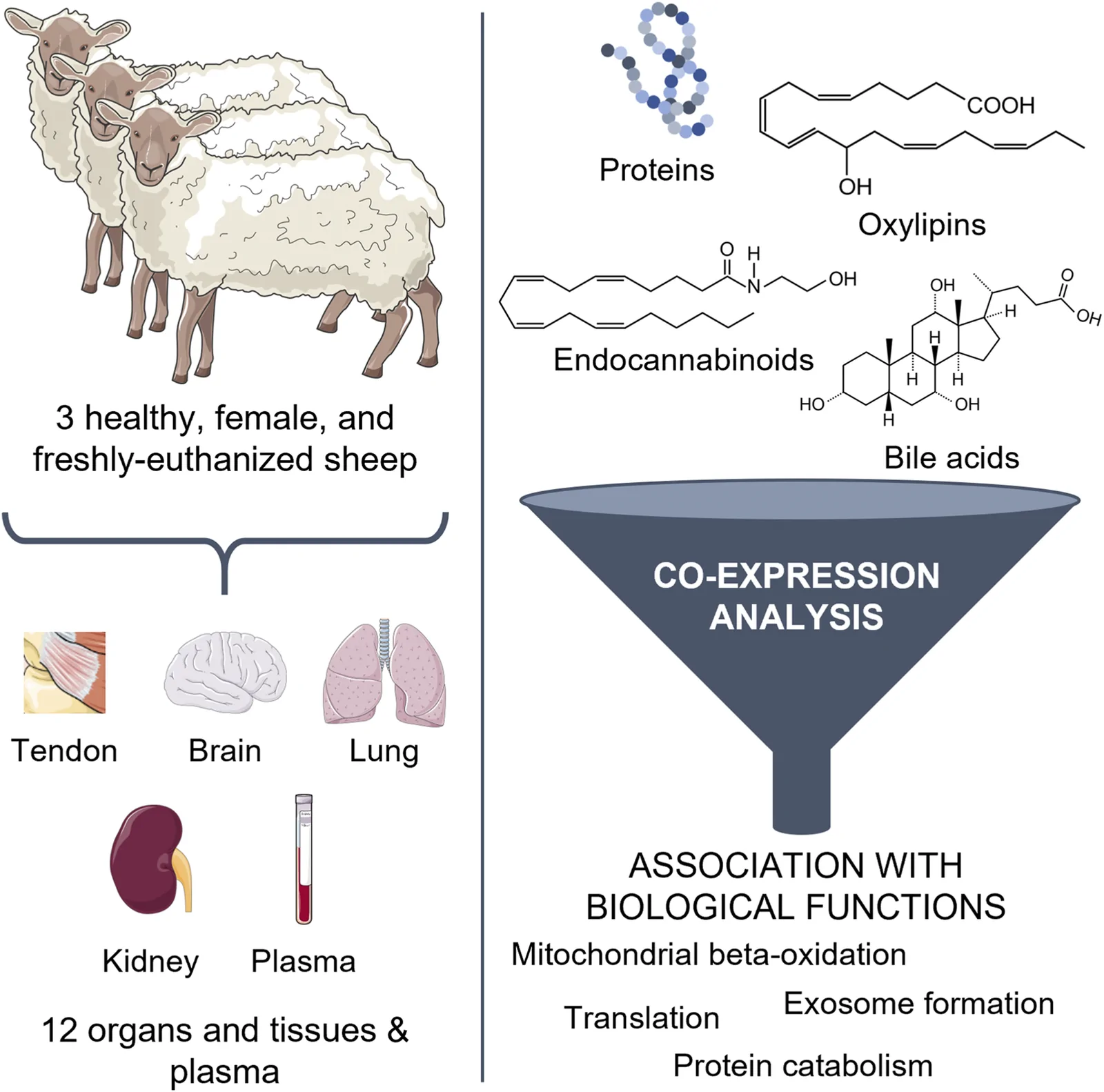

## 1 Introduction

Metabolic activities are known to be intricately linked with signaling and transcriptional networks that regulate cellular activities (Zhang et al., 2021; Zhu and Thompson, 2019; DeBerardinis et al., 2008). The metabolic profiles of cells change in response to substrate availability and throughout their lifespan to support their functional roles as they differentiate, age, or encounter stressors (Holcombe and Weavers, 2023). For instance, the metabolic activities in proliferating cells differ fundamentally from those in non-proliferating cells (Zhu and Thompson, 2019; Agathocleous and Harris, 2013). The tightly coordinated interplay between cellular physiology and metabolism is emerging as a fundamental determinant of tissue health and longevity and deserves further investigation (Sender and Milo, 2021; Ma et al., 2015; Banjac et al., 2023; Gerner et al., 2020). Actually, data on relevant metabolites and their functional implications remain scarce (Yan et al., 2023; Teder et al., 2023; Anand et al., 2022) and metabolomic studies have predominantly focused on tissues involved in energetically demanding tasks, such as physical work (*e.g.*, skeletal and cardiac muscles), neurological processes (brain), or vital physiological functions (liver, kidney), with little attention on bradytrophic tissues composed of largely quiescent cells such as cartilage and tendon.

Bioactive lipids, such as oxylipins, bile acids and endocannabinoids, are metabolites that may also act as signaling molecules, binding as ligands to specific receptors (Agathocleous and Harris, 2013; Wellen and Thompson, 2012; Gomes and Blenis, 2015; Murakami, 2011). Already more than 3 decades ago the term “oxylipins” was defined upon recognizing that a complex enzymatic system may exist which forms specific lipid mediators from polyunsaturated fatty acids (PUFAs) (Gerwick et al., 1991). This connection suggests that bioactive lipids may serve as indicators for the actual exertion of specific processes and may thus be used as biomarkers. The use of LC-MS/MS greatly supported the establishment of analytical platforms suitable for these molecules (Wolfer et al., 2015). While the use of pharmacological inhibitors and genetic approaches (Fujinaga et al., 2025; Cornejo-Garcia et al., 2016) were essential for oxylipin research, the integration of multi-omics represents a more recent and powerful approach to better understand physiological regulatory mechanisms (Cayer et al., 2026). Combining proteomics with bioactive lipid analysis, we have already described characteristic alterations of oxylipins and other bioactive lipids in human plasma samples in the course of an acute inflammatory challenge (Hagn et al., 2025). This motivated us to investigate the relation of these bioactive lipids with physiologic processes using this approach in a more systematic fashion.

The present study was designed accordingly and is based on two assumptions. First, different types of organs and tissues are associated with the exertion of specific and well-known biological processes, which affect both the proteome and the metabolome. Indeed, different tissues also exhibit distinct metabolic profiles (Muller et al., 2013). In adult mammals, physiological cell functions, but also the rate of cell proliferation and turnover, vary significantly between tissues, depending on each tissue’s specific function and activity status (Fang et al., 2023). The most characteristic biological processes associated with a given organ or tissue are strongly associated with the proteome, and accessible from experimental data *via* the gene ontology classification system (Ashburner et al., 2000). The second assumption of this study is that the abundance of a bioactive lipid or a molecular pattern of bioactive lipids detected in a tissue sample correlates with the activity status of a specific biological process. As a consequence, a correlation of biological processes, retrieved from proteomics data of tissue samples, with the pattern of bioactive lipids, detected in the same samples, across different organs and tissues should allow us to relate bioactive lipids with biological processes.

To that aim, this study determined the bioactive lipid fingerprints of plasma and 12 organs and tissues with differing metabolic activities (brain, heart, kidney cortex, renal medulla, liver, lung, muscle, nerve, skin, spleen, tendon, cartilage). The association of the occurrence of bioactive lipids with biological functions was investigated using an integrated mass spectrometry-based bioactive lipid and proteomics approach. We opted to utilize sheep (*Ovis aries*), a well-established large animal model, for this study. In contrast to small animal models such as mice, they are a long-lived species, and their body size, weight, and organ dimensions closely resemble those of humans (Ribitsch et al., 2021; Ribitsch et al., 2017). Already as early as in 1982, fundamental studies on the regulatory effects of oxylipins on the vascular tone have been performed in sheep (Schuette et al., 1982). However, while previous efforts have focused on the global analysis of gene expression across multiple ovine tissues, mass spectrometry (MS)-based proteome and metabolome data for the characterization of (ovine) tissues and organs with respect to molecules relevant for immune modulation hardly exist (Clark et al., 2017). We have established robust and reliable bioactive lipid and proteome profiling methods and have successfully combined these techniques for biomedical studies (Bortel et al., 2024; Kovarik et al., 2023; Janker et al., 2023). The poor functional annotation of sheep proteins by gene ontology was compensated using gene ontology annotations of human orthologues, previous work demonstrates this to work well (Ribitsch et al., 2021; Elkhamary et al., 2024). This facilitated the analysis of co-expression networks and clustering of the combined dataset as described previously (Mohr et al., 2021). This feasibility study resulted in numerous potential functional annotations of bioactive lipids which may help to better understand the physiological roles and potential disease implications of these important molecules.

## 2 Materials and methods

### 2.1 Sample acquisition and ethics

Organs and tissue were collected from three (n = 3), healthy, skeletally mature (2–4 years of age), female sheep (Merino) with a body weight between 70 and 90 kg which had been euthanized for reasons unrelated to this study. Euthanasia had been conducted following sedation with detomidine and butorphanol, placement of a catheter in the jugular vein, and induction of general anesthesia with thiopental, by the administration of T61, a veterinary euthanasia drug containing tetracaine hydrochloride, mebezonium iodide, and embutramide. Based on the “Good Scientific Practice, Ethics in Science and Research regulation” implemented at the University of Veterinary Medicine Vienna, the Institutional Ethics Committee and the Austrian Federal Ministry of Educations Science and Research, *in vitro* studies do not require approval if they are performed using tissues/organs which were obtained either solely for diagnostic or therapeutic purposes or in the course of other institutionally and nationally approved experiments.

Blood (5 mL) was collected from the jugular vein into ethylenediaminetetraacetic acid (EDTA) tubes and plasma was obtained *via* centrifugation (10 min, 4 °C, 2000 g) and stored at −80 °C until further analysis. In addition, samples were taken from twelve organs and tissues, the brain (caudal aspect of cerebrum), heart (apex), kidney cortex and renal medulla, liver (caudal lobe), lung (accessory lobe), muscle (m. gastrocnemius), nerve (brachial plexus), skin (lateral knee region), spleen, tendon (superficial digital flexor tendon of one front leg, mid tendon region) and cartilage (from the medial femoral condyle). For proteomics, about 5–10 mg of each organ/tissue were collected, whereas about 50–100 mg were collected for the bioactive lipid analysis. To remove blood cells as completely as possible, the obtained organ/tissue pieces were rigorously rinsed with ice cold phosphate-buffered saline (PBS) until no residual blood was visible on the sample surfaces. Care was taken that the organs/tissues were exsanguinated to the greatest possible extent, while the blood vessels and capillaries were not flushed. After washing, the tissue samples for proteomics analysis were immediately snap frozen in liquid nitrogen and stored at −80 °C. The samples for the bioactive lipid analysis were transferred into 15 mL Falcon™ tubes containing 1 mL of ice-cold ethanol (EtOH) and stored at −20 °C.

### 2.2 Proteomics and bioactive lipid sample preparation, data acquisition and processing

A detailed description of the following paragraphs on proteomics and bioactive lipid sample preparation, data acquisition and processing can be found in the Supplementary Material.

#### 2.2.1 Proteomics sample preparation

For untargeted tissue and plasma proteomics analysis, frozen tissue samples were transferred into 15 mL Falcon™ tubes containing 150 µL of lysis buffer (8 M urea, 50 mM triethylammonium bicarbonate (TEAB), 5% sodium dodecyl sulfate (SDS)) and homogenized using an ultrasound sonication homogenizer. After centrifugation at 5000 *g* and room temperature (RT) for 5 min, the supernatant was transferred into fresh tubes. Ovine plasma was diluted 1:20 using lysis buffer, and tissue and plasma samples were heated for 5 min at 95 °C and 300 rpm under constant shaking. After cooling to RT, the protein concentration was determined *via* a bicinchoninic acid (BCA) assay. Aliquots of plasma and tissue samples containing 20 µg protein were used for enzymatic protein digestion according to the ProtiFi S-Trap^TM^ protocol as described (Zougman et al., 2014).

#### 2.2.2 Proteomics data acquisition

Mass spectrometric analysis was performed with a timsTOF Pro (Bruker, Germany), in the Parallel Accumulation-Serial Fragmentation (PASEF) mode. The scan range was 100–1700 m*/z* for MS and MS/MS spectra recording and the 1/k0 scan range was set to 0.60–1.60 V*s cm^-2^ resulting in a ramp time of 100 m to achieve trapped ion mobility separation.

#### 2.2.3 Proteomics data processing

Protein identification was performed *via* MaxQuant (Cox and Mann, 2008) (version 1.6.17.0) employing the Andromeda search engine against the UniProt Database (Bateman et al., 2021) (version 11/2021, 20′375 entries). Search parameters were set as previously described (Weiss et al., 2021). Proteins with at least 60% identification rate in at least one group were considered for analysis. A two-sided Student’s t-test with S0 = 0.1 and an FDR cut-off of 0.05 was applied for identifying multiple testing-corrected significantly regulated proteins.

#### 2.2.4 Bioactive lipid sample preparation

For untargeted tissue and plasma analysis of oxylipins, fatty acids, bile acids, and other bioactive lipids, 400 mg EDTA-anticoagulated plasma was thawed on ice and added to a 15 mL Falcon™ tube containing ice-cold EtOH (1.6 mL, abs. 99.9%, AustrAlco) and an internal heavy isotope-labeled oxylipin standard mixture (Supplementary Table S1). All standards, including those indicated in Supplementary Table S2, were purchased by Cayman Chemical, Michigan, USA. The Falcon™ tubes containing tissue samples and ethanol were transferred from −20 °C to ice, homogenized using an ultrasound sonication homogenizer and stored again at −20 °C overnight. On the following day, liquid chromatography–mass spectrometry (LC-MS) grade water and the same internal standard mix that was used for the plasma samples were added, and all plasma and tissue samples were centrifuged. The supernatant was transferred, and the ethanol was evaporated. Solid phase extraction of tissue and plasma samples was performed as previously described (Leitner et al., 2022).

#### 2.2.5 Bioactive lipid data acquisition

For the LC–tandem mass spectrometry (LC-MS/MS) analyses, a Vanquish™ UHPLC system (Thermo Fisher Scientific™, Vienna, Austria) was coupled to a high-resolution quadrupole orbitrap mass spectrometer (Thermo Fisher Scientific™ Q Exactive™ HF hybrid quadrupole orbitrap mass spectrometer). Therefore, the Vanquish™ ultra-high performance LC (UHPLC) system was equipped with a reversed-phase Kinetex® XB-C18 column (2.6 µm XB-C18, 100 Å, LC column 150 × 2.1 mm, Torrance, CA, USA) to achieve analyte separation as previously described (Hagn et al., 2025). Thirty-three *m/z* values specific for oxylipins and their precursor fatty acids from an inclusion list were preferentially selected for fragmentation (Supplementary Table S3). The untargeted analysis of the following lipid main classes according to LIPID MAPS was carried out in negative ionization mode: oxylipins (octadecanoids, eicosanoids, and docosanoids), fatty acids and their conjugates, glycerophosphocholines, glycerophosphoethanolamines, sphingoid bases, and bile acids, while the following lipid main classes were acquired in positive ionization mode: fatty amides, monoradylglycerols, and steroids, as shown in Supplementary Table S2.

#### 2.2.6 Bioactive lipid data processing

For data analysis, raw files generated by the Q Exactive™ HF high-resolution mass spectrometer were evaluated using the TraceFinder software (version 4.1; Thermo Fisher Scientific™, Vienna, Austria) making use of measurements of purchased chemical standards (Supplementary Table S2). Additionally, MS/MS fragmentation spectra were evaluated referring to reference spectra of the commercially available standards or to reference spectra from the LIPID MAPS repository library (Fahy et al., 2007) using the Xcalibur™ Qual Browser software (version 4.1.31.9; Thermo Fisher Scientific™, Vienna, Austria). Coeluting isomers were reported giving the names of both analytes, *e.g.*, 12-HETE/8-HETE, as suggested by us and our colleagues of the oxylipin community (Schebb et al., 2025). The TraceFinder software (version 4.1; Thermo Fisher Scientific™, Vienna, Austria) was used for relative quantification allowing a mass deviation of 5 ppm as described before (Hagn et al., 2025). The resulting peak areas of each analyte with a S/N greater than 10 were exported and read using the R software package (version 4.2.0) (RStudio Team, 2020). The peak areas were log2-transformed and analyte peak areas were normalized to the internal standards to correct for variances arising from sample storage upon protein precipitation, extraction and LC-MS/MS analysis. Therefore, the log2-transformed mean peak area of the internal standards was subtracted from the log2-transformed analyte areas. The peak areas of the ovine plasma, organ and tissue samples were also normalized to the weight. Only molecules independently identified in all replicates of at least one tissue type were included in the statistical analysis. As several molecules turned out to occur at the limit of detection in other tissue types, data imputation was used to enable subsequent statistical analysis. To enable an imputation of missing values using the minProb function of the imputeLCMD package (version 2.1), 20 was added to the log2-transformed normalized areas to obtain values similar to proteomics label-free quantification (LFQ) values.

#### 2.2.7 Weighted co-expression network analysis (WGCNA) and semantic similarity analysis of the enriched gene ontology (GO) terms

After separate data preprocessing of the proteomics and bioactive lipid approaches, the resulting expression matrices were merged according to samples and evaluated by a weighted co-expression network analysis as previously reported by Langfelder et al. for weighted gene co-expression network analysis (WGCNA) (Langfelder and Horvath, 2008). An unsigned network was used with default parameters, except for β = 6, deepsplit = 4, Pearson correlation, minimal module size 30, pamStage = TRUE). The soft threshold (power) selection plots showing the scale independence and mean connectivity, which were used for the WGCNA, are provided in Supplementary Material - Supplementary Figure S1. Modules were associated with tissue types using the linear model ∼0 + TISSUE entered into the lmer package of R (Mohr et al., 2021) and subsequently set into biological context through GO term enrichment analysis after mapping UniProt IDs to gene symbols, using the *enricher* function from the clusterProfiler 4.0 R package with the Molecular Signatures Database (C5: Gene Ontology—Biological Processes) as input (Wu et al., 2021; Liberzon et al., 2011). Using the default parameters of the *pairwise_termsim* function from the clusterProfiler 4.0 R package, a Semantic Similarity Analysis of the enriched GO terms was generated, resulting in a hierarchical clustering thereof. The same package was also used to create dot and C-net plots (Wu et al., 2021).

#### 2.2.8 Data visualization

The extracted ion chromatogram was generated using the Xcalibur™ Qual Browser software (version 4.1.31.9; Thermo Fisher Scientific™, Vienna, Austria). Further data visualization was performed using the ggplot2 R package (Wickham, 2016). The graphical abstract was partly generated using Servier Medical Art, provided by Servier, licensed under a Creative Commons Attribution 3.0 Unported license.

#### 2.2.9 Data availability statement

Data derived from the bioactive lipid analysis and related metadata are available at the NIH Common Fund’s National Metabolomics Data Repository (NMDR) website, the Metabolomics Workbench, https://www.metabolomicsworkbench.org (accessed on 16 July 2026) (Sud et al., 2016), where they have been assigned to the following studies: Study ID ST003163 and ST003164. The DOI for this project (PR001968) is: http://dx.doi.org/10.21228/M8BF0G.

The mass spectrometry proteomics data have been deposited to the ProteomeXchange Consortium *via* the PRIDE (Perez-Riverol et al., 2025) partner repository with the dataset identifiers PXD064992 and PXD065008.

#### 2.2.10 Synthesis of the 4-HETE standard

A detailed description of the synthesis of the 4-HETE and the gas chromatography - MS (GC-MS) analysis results are provided in Supplementary Material.

## 3 Results

### 3.1 Identification and relative quantification of bioactive lipids, including the previously uncharacterized 4-HETE, in ovine plasma and 12 organ and tissue types

Taking account for the short half-life of many oxylipins, tissue samples were collected from anesthetized sheep and immediately snap frozen. The application of solid phase extraction to ethanol extracts of the homogenized sheep tissue samples allowed us to enrich various bioactive lipids comprising fatty acids and their conjugates, oxylipins (octadecanoids, eicosanoids, and docosanoids), lysolipids (glycerophosphocholines and glycerophosphoethanolamines), endocannabinoids and other fatty amides, sterol lipids such as bile acids or the steroid cortisol, the monoradylglycerol 2-arachidonoyl-glycerol, and the sphingoid base sphingosine-1-phosphate. In addition, the applied anesthetic embutramide was also detected in all samples. Using high-resolution mass spectrometry and 114 analytical standards for verification, 168 distinct molecules were reliably and reproducibly identified in all three replicates of at least one organ or tissue type (Supplementary Table S2, the level of identification is indicated for each molecule). For comparative analysis, the determined abundance values (area under the curve values) were normalized using a set of heavy isotope-labeled oxylipins as internal standards.

Additional 40 molecules were identified as oxylipins with uncertain identity. Due to high structural ambiguity of oxylipin isobars and a lack of matching analytical standards, it was not yet possible to assign unambiguous structures to these molecules. These oxylipins were presently designated according to the formula “molecular mass”_“chromatographic retention time”. One of those was subsequently identified by us as 4-hydroxy-eicosatetraenoic acid (4-HETE, Figure 1), as outlined in more detail in the following. The extracted ion chromatogram illustrates the chromatographic separation of various hydroxy-eicosatetraenoic acids (HETEs) and isomeric epoxy-eicosatrienoic acids, which differ with regard to the position of the hydroxy- or epoxy-group within the molecule. The identification of 5-HETE, 8-HETE, 9-HETE, 11-HETE, 12-HETE and 15-HETE as well as 11(12)-epoxy-eicosatrienoic acid (EET) and 14(15)-EET was successfully verified by analytical standards, as they displayed the same chromatographic retention times, exact molecular mass, isotopic pattern and fragmentation patterns. However, no analytical standard was commercially available for this unknown oxylipin, which as presently identified in heart, kidney cortex, liver, lung, muscle and spleen tissue (Figure 1). Actually, the exact molecular mass and the assignment of specific fragment ions as indicated in Figure 1 suggested a putative identification as 4-HETE. The MS/MS fragmentation pattern thereof, characterized by the specific fragment of *m/z* = 101.024, is a well-known marker of oxylipins with a functional group at position C4 such as the docosahexaenoic acid (DHA)-derived products 4-hydroxy-docosahexaenoic acid (HDoHE or HDHA) (Supplementary Material - Supplementary Figure S2) or 4-F4t-Neuroprostane (LIPID MAPS–Standards Spectra Database; LMFA04010524) (Cockayne et al., 2026). Subsequently, we were able to verify this putative identification *via* chemical synthesis and LC-MS analysis thereof. The 4-HETE synthesis protocol and analytical verification *via* GC-MS is described in detail in the Supplementary Material. This oxylipin was previously also not listed in LIPID MAPS, a database of well characterized lipids with largely known biochemistry (Fahy et al., 2007). Upon sharing the analytical standard of 4-HETE with LIPID MAPS by us, it has now been listed (LMFA03060123). Evidently, additional effort needs to be spent for the characterization of potential physiologic effects of this previously undescribed oxylipin, as well as the identity and potential functions of the other oxylipins lacking structural identification.

**FIGURE 1:**
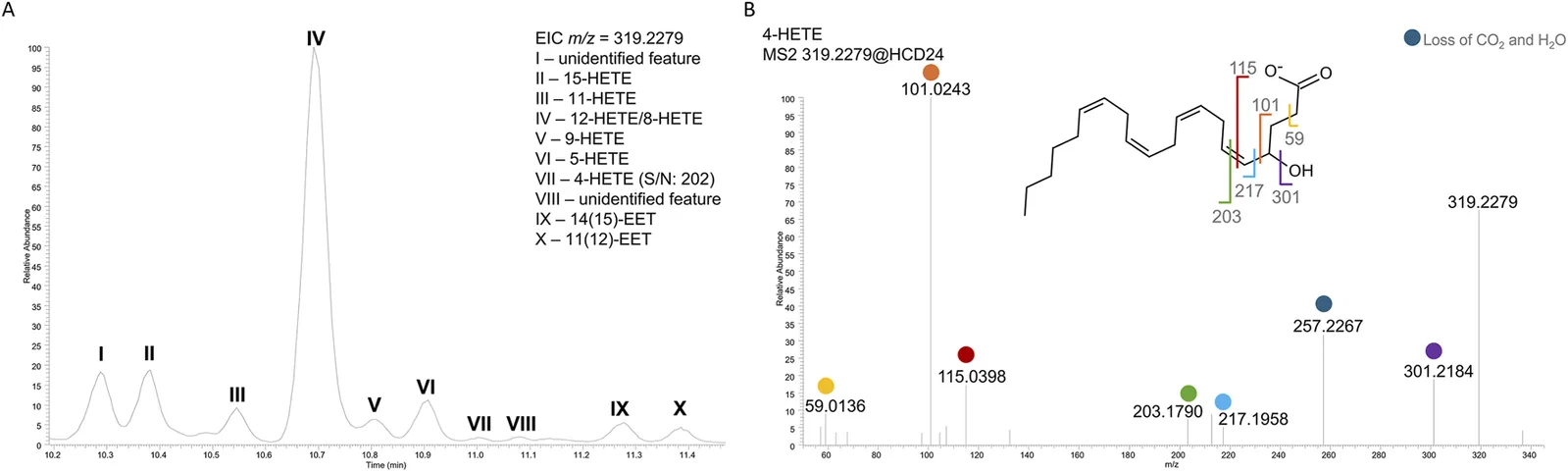
Identification of the previously uncharacterized oxylipin 4-HETE by LC-MS/MS. **(A)** Extracted ion chromatogram (EIC) for *m/z* = 319.2279 obtained from a lung sample. **(B)** MS2 spectrum obtained for peak VII (S/N: 202) indicated in the EIC and the assignment of the identifying molecular fragments according to the molecular structure of 4-HETE.

### 3.2 Bioactive lipids display a tissue-specific expression pattern

While some molecules such as deoxycholic acid showed low specificity with regard to organ and tissue types (Figure 2A), others were exclusively identified in one or a few thereof. This is exemplified for DHA isoform I, representing DHA with one *trans*-configured double bond as described previously (Leitner et al., 2022), which was detected only in plasma (Figure 2B).

**FIGURE 2:**
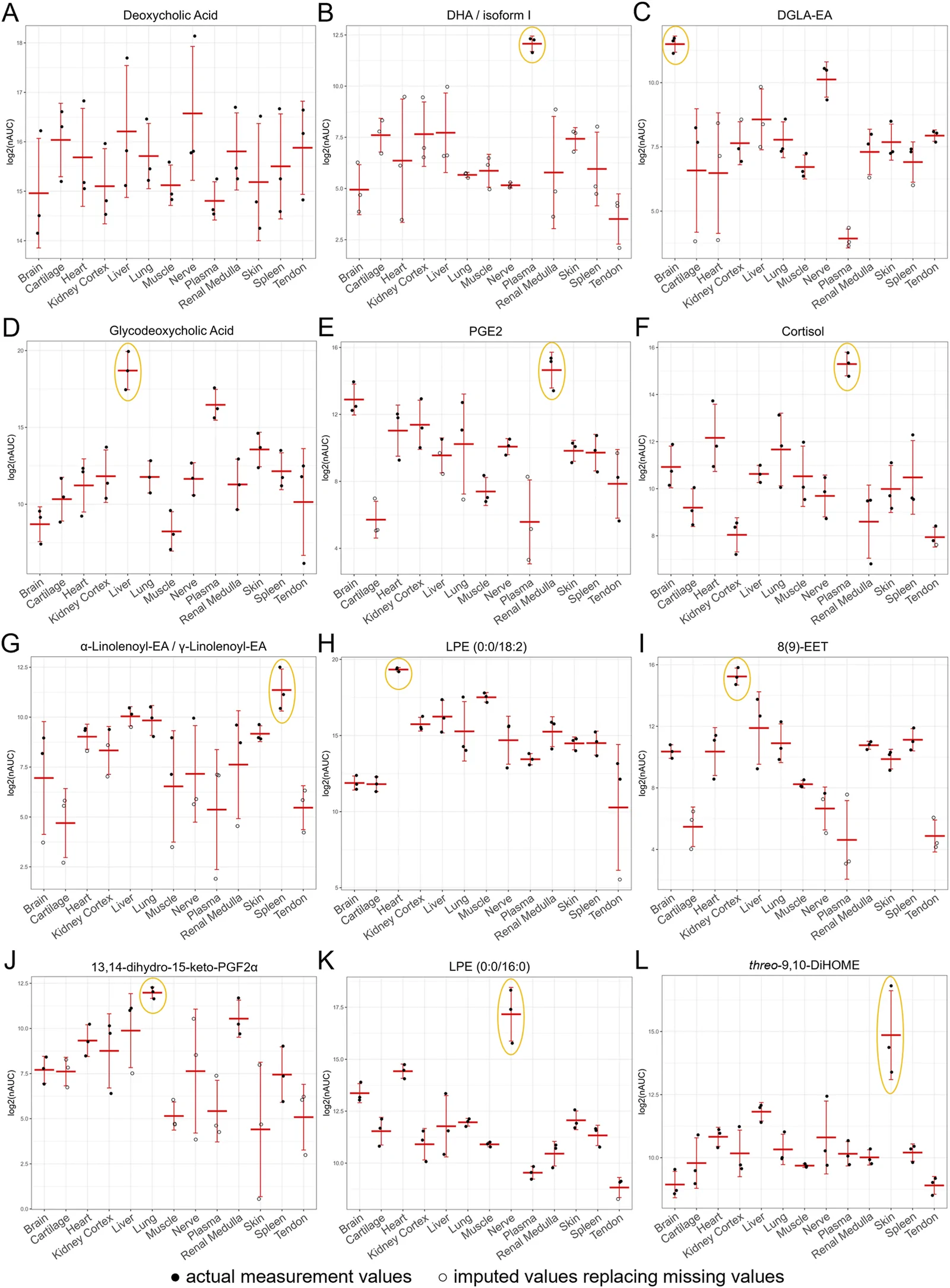
Relative abundance values, i.e., normalized area under curve (nAUC), of selected molecules showing low specificity (**(A)** deoxycholic acid), high specificity (**(B)** DHA isoform I, one double-bond shows *trans*-configuration, note that all values but for plasma are imputed) or a predominant occurrence in one organ or tissue type of **(C-L)**: DGLA-EA, Glycodeoxycholic Acid, PGE2, Cortisol, Alpha-Linolenoyl-EA/gamma-Linolenoyl-EA, LPE (0:0/18:2), 8(9))-EET, 13,14-dihydro-15-keto-PGF2alpha, LPE (0:0/16:0), and *threo*-9,10-DiHOME. Full circles, representation of the actual measurement result; open circles, imputed values replacing missing values (indicating lack of detection).

Numerous expression patterns characteristic for various tissue types were observed, as depicted in Figures 2B–L. Several of those observations are in accordance with known bioactive lipid functions as listed in the following. The endocannabinoid dihomo-gamma-linolenoyl ethanolamide (DGLA-EA) was predominantly found in the brain (Figure 2C) (Pertwee, 2015), the bile acid and nutrient signaling hormone glycodeoxycholic acid (Zhou and Hylemon, 2014) predominantly in liver (Figure 2D), the prostaglandin (PG) and renal water transport regulator PGE2 (Li et al., 2017) in renal medulla (Figure 2E) and the stress hormone cortisol (Benfield et al., 2014) in plasma (Figure 2F). Alpha-linolenoyl ethanolamide, predominant in spleen tissue (Figure 2G), may relate to its physiological role as immune modulator for macrophages (Ishida et al., 2013). Remarkably, the present analysis also revealed characteristic patterns of molecules with yet unknown functional implications. This included the lyso-phosphatidylethanolamine (LPE) (0:0/18:2) found to be predominant in the heart (Figure 2H), 8(9)-EET in the kidney cortex (Figure 2I), 13,14-dihydro-15-keto-PGF2alpha in the lung (Figure 2J), LPE (0:0/16:0) in nerve tissue (Figure 2K), and *threo*-9,10-dihydroxy-octadecenoic acid (DiHOME) in the skin (Figure 2L).

### 3.3 Levels of free PUFAs and PUFA-derivatives correlate between organ types

As several immune modulatory molecules are derived from PUFAs, relative abundance values of the main PUFAs arachidonic acid (AA) and DHA were closely inspected. As demonstrated in Figure 3, high levels of PUFAs were detected in the kidney cortex, liver and brain, intermediate levels in the lung, nerve tissue, skin, spleen and renal medulla, and the lowest levels in cartilage, heart, muscle, and tendon tissues (Figures 3A,D). The levels of the corresponding PUFA-containing lyso-phosphatidylcholine (LPC) molecules (Figures 3B,E) and endocannabinoids (Figures 3C,F) were found to correlate with those basal PUFA levels. However, the comparative analysis demonstrated that some molecules escaped this pattern. For example, the levels of LPC (0:0/20:4) in heart tissue were higher than expected from the levels of the 20:4 fatty acid AA in heart tissue (Figure 3B), whereas LPC (0:0/22:6) in heart tissue (Figure 3E) was as close to the 22:6 fatty acid DHA (Figure 3D) as in other tissues. This specific pattern strongly suggested tissue-specific metabolic processes and motivated us to perform more systematic association analyses.

**FIGURE 3:**
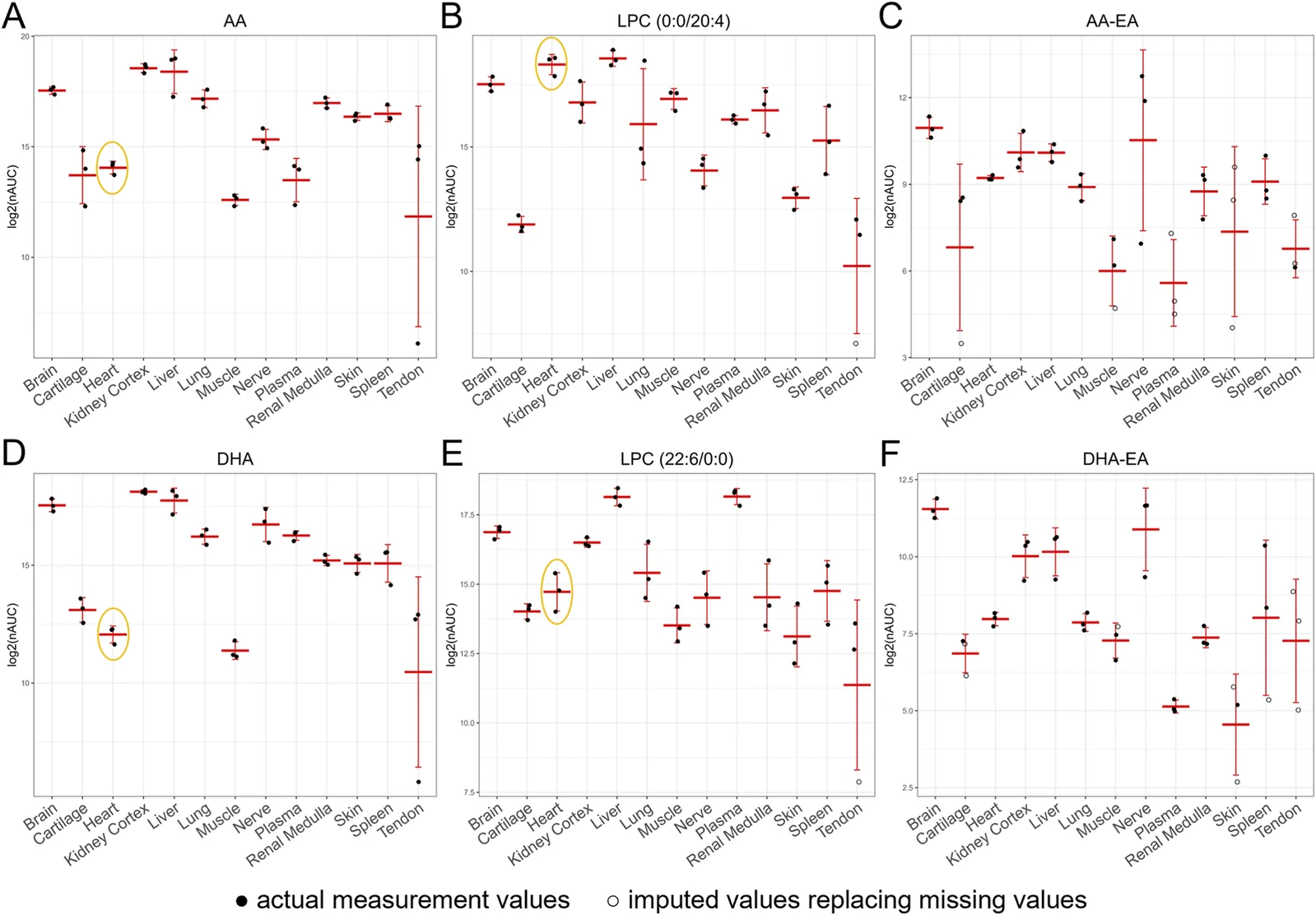
Relative abundance values, i.e., normalized area under curve (nAUC), of PUFA-related molecules derived from AA **(A-C)** and DHA **(D-F)**. Note that the expression patterns of these related molecules correlate in most tissues, characteristic deviations as indicated in case of LPC (0:0/20:4) may thus indicate specific metabolic processes.

### 3.4 Association analysis of bioactive lipids indicates functional correlations

A heatmap resulting from an association analysis of the bioactive lipids detected in the tissue and organ samples is displayed in Figure 4. As expected from the expression pattern depicted in Figure 3, members of the same lipid class, including fatty acids, bile acids, lysolipids, endocannabinoids and oxylipins, were found to cluster (Figure 4, cluster and color-coded bar on the left-hand side). The anesthetic embutramide used to euthanize the sheep was included in the analysis, while it is no endogenous metabolite. However, the relative abundance differences illustrate the characteristic properties of tissue types regarding the accumulation of this drug. Remarkably, embutramide was hardly detectable in plasma, indicating a fast and highly efficient accumulation in the heart, lungs and brain.

**FIGURE 4:**
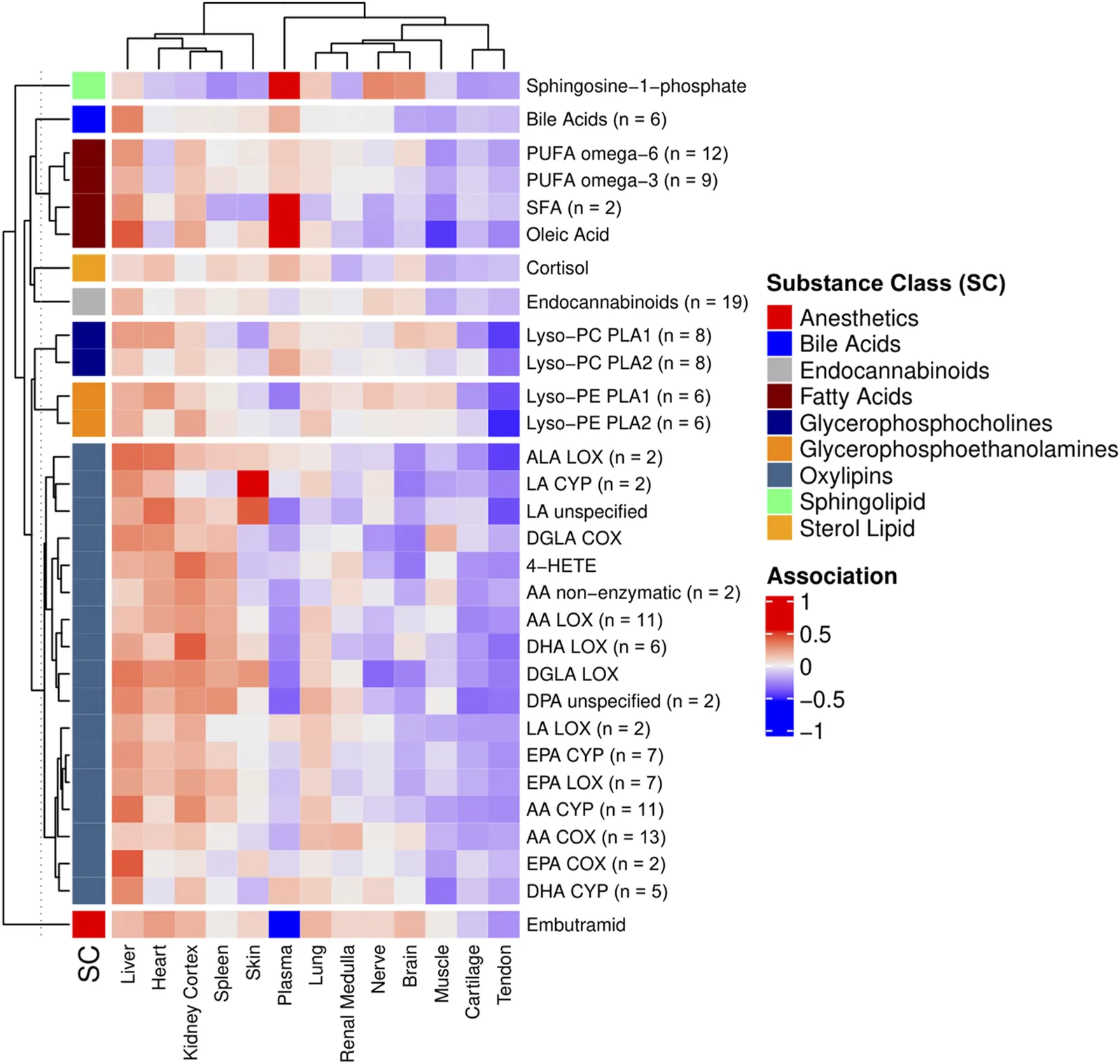
Association analysis of bioactive lipids. Hierarchical clustering grouped all oxylipins into a large cluster, oxylipin subclasses were annotated according to the precursor fatty acids: alpha-linolenic acid (ALA), linoleic acid (LA), dihomo-gamma-linolenic acid (DGLA), arachidonic acid (AA), docosahexaenoic acid (DHA), docosapentaenoic acid (DPA) and eicosapentaenoic acid (EPA); and the associated enzymes: lipoxygenase products (LOX), cytochrome p450 products (CYP), cyclooxygenase products (COX), or non-enzymatic. The lysolipids were classified according to phospholipase A1 (PLA1) or phospholipase A2 (PLA2) products. The fatty acids were classified as saturated fatty acids (SFA), omega-3 and omega-6 polyunsaturated fatty acids (PUFAs).

The cluster analysis actually formed two main tissue cluster separated from plasma. Apparently one group represented tissue with rather high levels of bioactive lipids, in contrast to the other with rather low levels (Figure 4, cluster on the top). While heart tissue belonged to the first cluster, skeletal muscle tissue was in the second, indicating profound differences of lipid metabolism between these two muscle tissue types.

### 3.5 Functional characterization of tissue types by proteome profiling

Samples from the solid tissue types and plasma were subjected to proteome profiling as described previously (Elkhamary et al., 2024). After filtering for reproducible and highly confident results as described in the Methods section, between 171 (cartilage) and 2761 (brain) proteins were identified per tissue type, resulting in a total of 4717 proteins covering a dynamic range of label free quantification values of five orders of magnitude (Supplementary Table S4). Mutual comparisons of each tissue type against the sum of all other tissue types identified proteins specifically enriched in one tissue/organ type. The numbers ranged from 461 proteins found to be enriched in brain tissue brain down to only two proteins found to be enriched in tendons (red marked proteins, Supplementary Table S4). These proteins comprised many well-known tissue-specific human orthologues as characterized by gene ontology, again qualifying this approach.

Gene ontology (GO) term enrichment analysis was used for functional characterization of organ types as depicted in Figure 5 in case of brain tissue. Processes such as “neuron projection” and “intracellular signal transduction” were found to be over-represented in brain tissue. The presynaptic membrane, synaptic vesicle and synapse were identified as hub-terms linking a large number of characteristic proteins (Figure 5). Thus, characteristic assignment of tissue-specific functions *via* the proteomics data was obtained and allowed us to correlate proteins mediating characteristic tissue functions with bioactive lipids.

**FIGURE 5:**
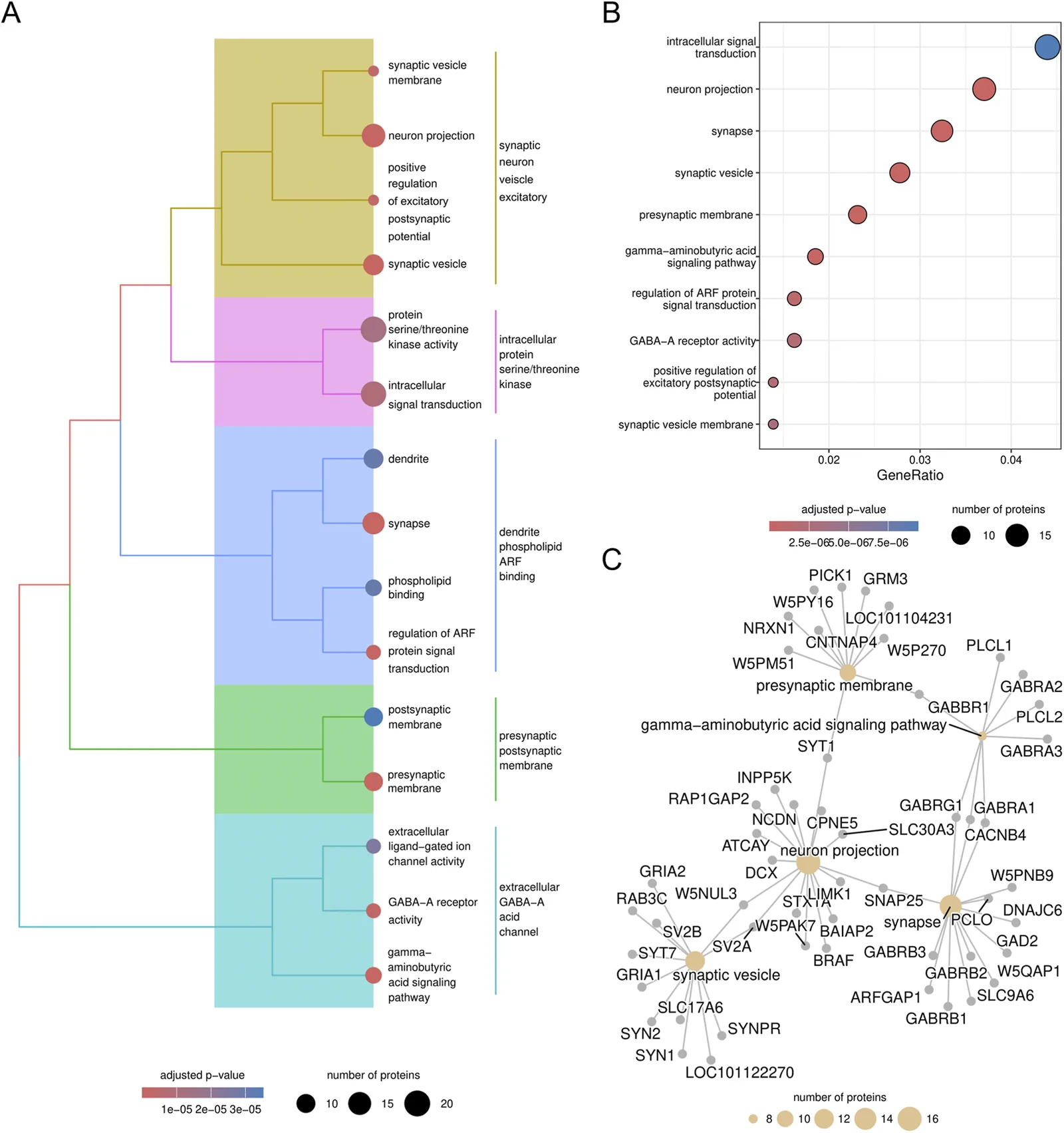
Results of the GO term enrichment analysis obtained from proteome profiling of ovine brain tissue as **(A)** tree plot of the hierarchical cluster analysis of the enriched GO terms, **(B)** dot plot of the 10 most significantly enriched GO terms and **(C)** C-net plot of a network of the five most significantly enriched GO terms in the ovine brain tissue. Dot size indicates the number of participating proteins in the respective GO biological process. Dot color indicates the enrichment test adjusted p-value **(A,B)**.

### 3.6 Principal component analysis (PCA) of proteome profiles compared to bioactive lipid profiles

To assess the overall structure of the proteomics and bioactive lipid analysis datasets, principal component analysis (PCA) was performed for the respective datasets. Technical replicates from the bioactive lipid analysis were also included in the corresponding PCA. Both the PCA of the proteomics label-free quantification (LFQ) values (Figure 6A) and the PCA of the normalized area under the curve (nAUC) values of the bioactive lipid analysis (Figure 6B) show clear clusters according to the organ or tissue types. As expected from the positive identification of numerous tissue-specific proteins compared to fewer lipids with tissue-specific expression patterns, the clusters observed *via* proteome analysis results were better separated. Muscle, cartilage, and tendon clustered away from other tissue types in both PCAs. Remarkably, brain and nerve tissue were clearly separated on the proteome level, while clustering together on the level of bioactive lipids. On the other hand, the separation of brain and liver clusters *via* bioactive lipids was more distinct than the separation *via* proteins.

**FIGURE 6:**
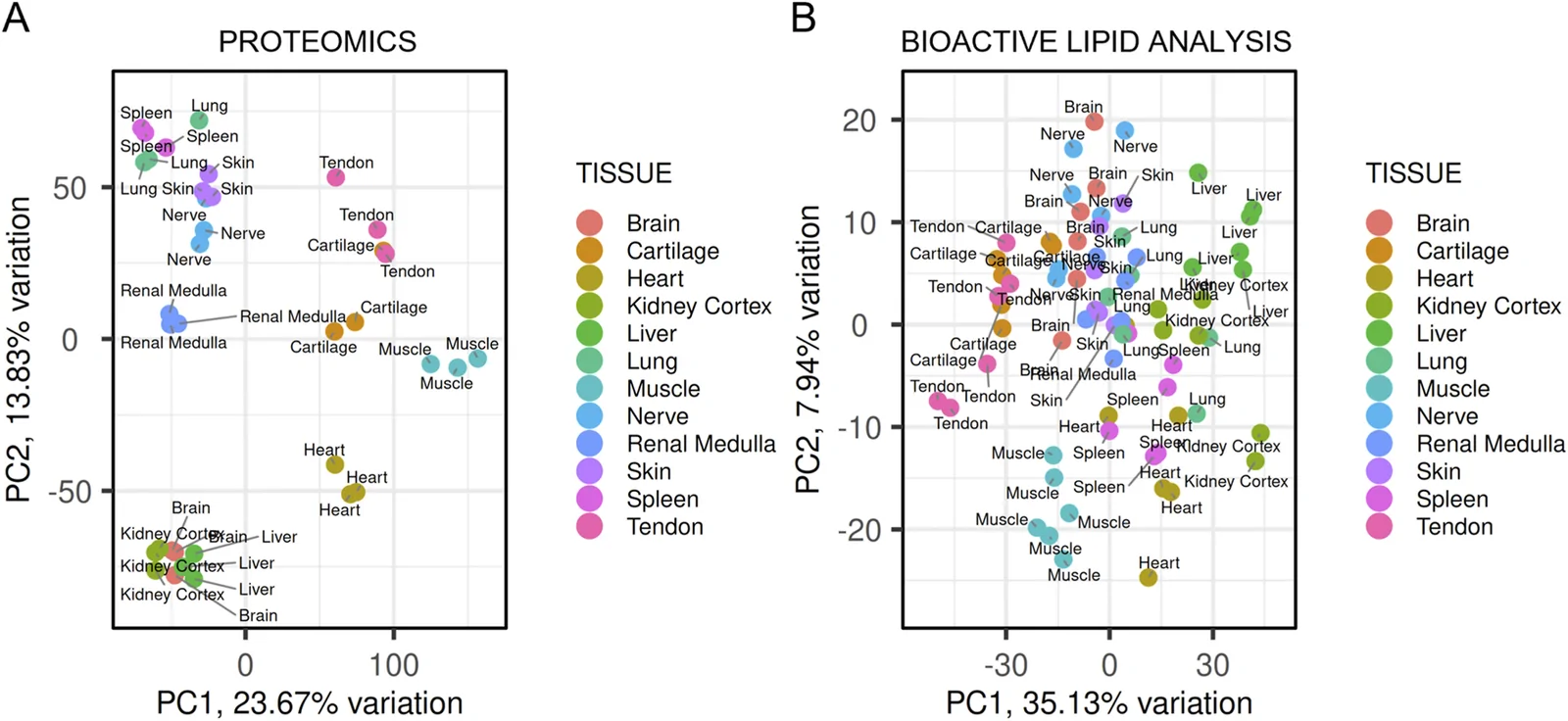
Principal component analysis (PCA) of **(A)** proteomics label-free quantification (LFQ) values and **(B)** bioactive lipid normalized area under the curve (nAUC) values for 12 organ and tissue types collected from three sheep. Technical replicates are also provided for the bioactive lipid PCA.

### 3.7 Co-expression analysis of bioactive lipids and proteins reveals functional correlations

Co-expression analysis of all identified bioactive lipids with all identified proteins and clustering thereof resulted in the formation of 27 modules (Supplementary Table S5). The efficient separation of modules is demonstrated in Supplementary Figure S3 (Supplementary Material). These modules showed pronounced tissue-type specific characteristics as depicted in Figure 7. A table of the corresponding p-values and the t-statistic values of the organ and tissue types of each module can be found in Supplementary Table S6. To give examples, module M12 was most representative for renal medulla, module M15 for kidney cortex, module M24 for nerve tissue. On the other hand, some modules showed characteristic under-representation, such as module M14 in case of cartilage and tendon tissue (Figure 7). While nine modules listed proteins only, 18 modules successfully correlated proteins with bioactive lipids, allowing for positive or negative correlations. Investigation of proteins listed in the modules by gene ontology term enrichment analysis allowed us to relate a given set of bioactive lipids with cellular functions characteristic for the given tissue type (Supplementary Table S5).

**FIGURE 7:**
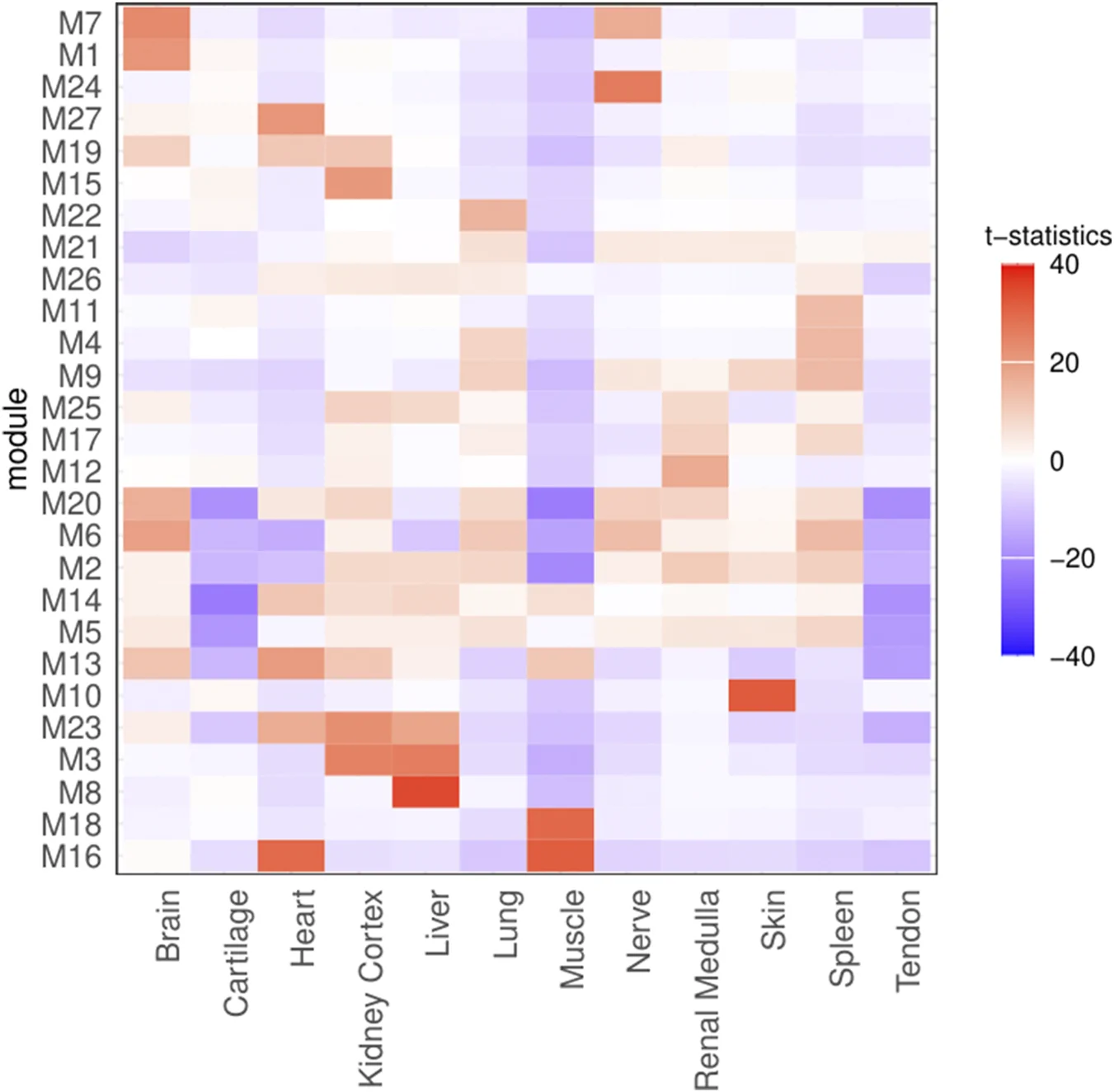
Heatmap of Eigengene analysis of the 27 modules. Several modules such as M1, M8 and M18 were found apparently specific for one organ type, others such as M3 and M16 were characteristic for two tissue types, whereas other modules such as M5 and M14 displayed characteristic negative correlations.

In order to evaluate whether the obtained results contained reliable information, the modules were screened for correlations compatible with established knowledge. Module M8 provided a positive example, correlating the CYP monooxygenase products 11,12-dihydroxy-eicosatrienoic acid (DiHETrE), 14,15-dihydroxy-eicosatetraenoic acid (DiHETE), 16,17-dihydroxy-docosapentaenoic acid (DiHDPA) and 19,20-DiHDPA, with a cluster of monooxygenases (CYP1A2, CYP2A13, CYP4F2, CYP4F3, CYP8B1, FMO1, FMO5; Supplementary Table S5). Module M8 was also found to be highly specific for the liver (Figure 7), demonstrating the liver-specific expression pattern of the monooxygenases (Leroux et al., 1989) and correctly correlating them with their reaction products. Thus, the information retrieved from module M8 reproduced successfully established biochemical knowledge and demonstrated that the correlation analysis worked as expected.

Subsequently, the modules were evaluated with regard to potential new findings. Module M2 correlated low levels of PUFAs - including DHA, DPA_ω-3_, DPA_ω-6_, EPA, DGLA, and AA- and mainly CYP derivatives thereof (*e.g.*, 14(15)-EET, 14,15-DiHETrE, 20-HDoHE, 20-HETE, 20-carboxy-arachidonic acid (20-COOH-AA), 22-HDHA, and the novel characterized 4-HETE) with low levels of proteins involved in cytoplasmic translation and endoplasmic reticulum function. This module was characteristic for liver, lung, and kidney, but showed a characteristic negative correlation in muscle, tendon and cartilage. Module M3 correlated the less unsaturated or fully saturated fatty acids oleic acid, linoleic acid and palmitic acid together with 18-HETE and 19-HETE with high levels of proteins involved in mitochondrial fatty acid beta-oxidation. Module M3 was mainly characteristic for kidney cortex and liver. Module M1, apparently specific for brain, correlated 11,12-DiHETE, 15-deoxy-PGJ2 and 5(6)-EET with proteins mediating chemical synaptic transmission. Module M18 correlated arachidonoyl ethanolamide phosphate with proteins essential for muscle contraction. Module M26 correlated numerous LPCs and LPEs together with several oxylipins with proteins characteristic for extracellular exosomes.

Other modules clustered proteins with characteristic functional relations, such as module M17 associated with mRNA splicing, module M21 associated with extracellular matrix components and module M22 associated with angiogenesis, but did not show any significant correlations with bioactive lipids.

## 4 Discussion

Here we present a comprehensive analysis of bioactive lipids of twelve different tissues and organs and plasma from healthy adult sheep, Ovis aries. The bioactive lipids comprise different classes of lipids, including fatty acids and their conjugates, oxylipins (octadecanoids, eicosanoids, and docosanoids), lysolipids (glycerophosphocholines and glycerophosphoethanolamines), sphingoid bases, monoradylglycerols, fatty amides such as endocannabinoids, and sterol lipids like bile acids and steroids. Furthermore, these lipid profiles were combined with corresponding proteome profiles of the same tissue and organ samples. While hardly any tissue-specific bioactive lipids were identified fulfilling the same stringent specificity criteria as applied for tissue-specific proteins, bioactive lipid patterns characteristic for tissues were clearly discerned (Figure 2). To the best of our knowledge, this is the first study systematically investigating tissue and organ expression characteristics of bioactive lipids in relation to proteins. By carefully sampling freshly isolated tissue samples from euthanized animals, we were able to obtain reliable expression pattern of molecules which may otherwise be associated with methodological obstacles (Schebb et al., 2022). We have previously described the deregulation of eicosanoids and other bioactive lipids in the context of chronic inflammation (Tahir et al., 2017), ovarian cancer (Muqaku et al., 2020) and heart diseases such as functional mitral regurgitation (Hofbauer et al., 2023) and clearly demonstrated the power of oxylipins at nanomolar concentrations for the modulation of inflammatory processes in cell culture experiments (Niederstaetter et al., 2021). Using a data-dependent acquisition method, we presently identified the previously undefined oxylipin 4-HETE (Figure 1). The reliable identification of this arachidonic acid (AA)-derived compound was verified *via* chemical synthesis. Since AA lacks a double bond between C4 and C5 for potential autoxidation, it is most plausible that it may be formed *via* an allylic hydroxylation by CYP *in vivo* (Quaranta et al., 2022).

Clustering the tissue types according to the bioactive lipid profiles separated three main tissue clusters, *i.e.*, high levels versus low levels of bioactive lipids and plasma (Figure 4). Unexpectedly, heart muscle and skeletal muscle did not cluster together. Heart tissue, belonging to the high lipids level cluster, was described to have high levels of lipoprotein lipase in contrast to skeletal muscle (Zierler et al., 2013), suggesting higher lipid turnover rates and thus higher levels of bioactive lipids. Thus, this observation is consistent with previous knowledge and points to important metabolic characteristics of these otherwise rather similar tissue types.

The anesthetic embutramide used to euthanize the sheep was included in the analysis, while it is no endogenous metabolite. While the drug was supplied by blood, plasma levels were much lower than tissue levels only a few minutes after drug application. These relative abundance differences may thus illustrate the redistribution dynamics of the pharmacologic substance, highlighting the different access to blood supply and drug affinity properties of tissue types. Figure 4 illustrates a highly efficient uptake in the heart, lungs and brain in contrast to rather poor uptake in muscle, tendon and cartilage.

Most bioactive lipids were detected in almost all samples, indicating their ubiquitous presence. As suggested by clustering, the bioactive lipid composition of plasma differed profoundly from that of solid organs and tissues (Figure 4). This observation is also consistent with established knowledge, as plasma lipids mainly derive from lipoprotein particles, whereas tissue lipids mainly derive from cell membranes and lipid stores. As many bioactive lipids have been described to be rather short-lived (Wymann and Schneiter, 2008), plasma levels of such bioactive lipids may quickly fall below detection thresholds. This seems to apply, *e.g.*, to the prostaglandin PGE2, described to be almost completely degraded within 1 minute in plasma (Hamberg and Samuelsson, 1971). In this study, PGE2 was identified in all tissue types but cartilage and plasma (Figure 2), indicating that the presently applied sample preparation protocol allowed us to preserve meaningful oxylipins while being short-lived. This finding indicates continuous PGE2 synthesis in the healthy organs for the maintenance of physiologic processes such as water transport in renal collecting ducts (Li et al., 2017). However, increased plasma levels of PGE2 may relate to chronic inflammation. Plasma levels of PGE2 were found increased in human patients suffering from inflammatory bowel disease (Janker et al., 2023) and type II diabetes (Fenske et al., 2022). To sum up, the present data strongly suggest that several bioactive lipids presently detected in organs and tissues may have relevant, but hardly characterized, physiological functions and may serve as biomarkers for the corresponding processes.

In order to learn more about such physiological functions, we have conducted a correlation analysis of bioactive lipids with proteins, making use of the ample functional annotation of proteins *via* gene ontology (Ashburner et al., 2000). As most bioactive lipids were detected at varying amounts in the different organ and tissue types, this approach promised to deliver reasonable functional correlations. Indeed, monooxygenase products clustered with the corresponding enzymes, the monooxygenases, in a module characteristic for liver tissue. In addition, the module M3, characteristic for the kidney cortex, contained 18-HETE and 19-HETE together with proteins involved in mitochondrial beta-oxidation (Figure 7). At one hand, this is consistent with the literature, as both HETEs have previously been detected in the kidneys, and 19-HETE has been described to be essential for the stimulation of proximal renal tubules responsible for the reabsorption of water, ions, and nutrients–an energy-consuming process enabled through the oxidative metabolism *via* abundant mitochondria present in these tubules (Shoieb et al., 2019; Tang et al., 2021). On the other hand, the present analysis suggests a previously undescribed functional relation between 18-HETE and mitochondrial beta-oxidation contributing to proximal renal tubule function. The Eigengene analysis depicted in Figure 7 highlighted another correlation between the oxylipins 11,12-DiHETE, 15-deoxy-PGJ2 and 5(6)-EET and chemical synaptic transmission in the ovine brain (module M1, Figure 7). This finding is also supported by independent previous observations, as these oxylipins have indeed been described to occur in the brain or the central nervous system (Broos et al., 2023; Iliff et al., 2010; Mouihate et al., 2004), while 5(6)-EET seems to be directly involved in synaptic transmission (Sisignano et al., 2012), and 15-deoxy-PGJ2 is a potent modulator of this physiological process (Costello et al., 2005).

A potentially new but highly relevant finding is the correlation of PUFAs with endoplasmic reticulum and ribosomal proteins, all of which are involved in protein synthesis. The functional implications of PUFAs have been extensively investigated, indicating that, *e.g.*, high levels of PUFAs may support the function of the blood-brain barrier and cardiovascular health, potentially due to anti-inflammatory properties (Wen et al., 2024; Endo and Arita, 2016). The high content of PUFAs in cardiolipins, essential for appropriate mitochondrial functions, led to the suggestion that PUFAs may correlate with mitochondrial abundance (Stanley et al., 2012). However, the present data demonstrate that high levels of mitochondrial proteins in muscle and heart were associated with low levels of PUFAs, apparently contradicting this assumption. PUFAs were presently found to be most abundant in three tissue types, namely in brain, liver, and kidney cortex (Figure 3), hardly compatible with this suggestion. Furthermore, longevity and proper immune cell functions were suggested to correlate with high levels of PUFAs (Qiu et al., 2024; Miles et al., 2021). Nevertheless, neither longevity as attributed to brain cells, nor high levels of immune cells as evident in the case of the spleen, nor mitochondrial abundance as evident for muscle tissue are common properties of the three tissue types high in PUFAs. However, high levels of membrane-rich endoplasmic reticulum are indeed a common characteristic of tissues with high protein turnover. The chaperoning function of the endoplasmic reticulum (ER) has been described to be critically dependent on lipids showing a broad range of chemical diversity, thus also rich in PUFAs (Bogdanov and Dowhan, 1999). A specific support of chaperoning functions by PUFA-containing endoplasmic reticulum membrane lipids may thus explain the former as well as present observations, and thus suggests an updated model for the functional role of PUFA-containing lipids with intriguing implications.

Tissue types with little protein synthesis and turnover such as cartilage, muscle and tendon were found to contain low levels of PUFAs and the corresponding, potentially anti-inflammatory oxylipins, designated as pro-resolving mediators (Julliard et al., 2022). Indeed, pro-resolving mediators, including the AA-derived EETs, have been described to mediate efferocytosis, the removal of dead cells and debris (Fredman et al., 2023; Gilroy et al., 2016). Next to EETs, further CYP-derived oxylipins such as 20-HETE or 20-COOH-AA correlating with protein synthesis and turnover have been described as anti-inflammatory agents *via* activation of PPAR α and γ (Gilroy et al., 2016; Fang et al., 2007). Interestingly, the novel characterized AA-derived oxylipin 4-HETE was also associated with protein synthesis and turnover, and may have anti-inflammatory potential as well. A lack of omega-3 fatty acid-derived bioactive lipids, apparently also associated with ageing, may thus limit efferocytosis and drive chronic inflammation (Panigrahy et al., 2021; Lamon-Fava, 2025). In line, *in vivo* data demonstrated the importance of PUFAs for the exertion of phagocytosis by macrophages in atherosclerotic mice (Li et al., 2009). As a consequence, low levels of omega-3 and omega-6 fatty acid-derived pro-resolving bioactive lipids in cartilage, muscle and tendon seem to be associated with an inherent risk for chronic inflammatory diseases such as osteoarthritis or tendinopathies. Actually chronic inflammation is a characteristic feature of most age-associated health threats including cardiovascular diseases, diabetes, osteoarthritis, cancer and neurodegenerative diseases (Freund et al., 2010). The increased risk for such diseases with age may thus be a consequence of declining protein and lipid turnover (Basisty et al., 2018; Johnson and Stolzing, 2019), and increased protein misfolding (Relini et al., 2013), eventually resulting in inflammasome activation and sustained inflammation (Zhu et al., 2014).

Protein chaperoning and replacement of damaged protein *via* synthesis may thus synergize with anti-inflammatory properties of PUFA derivatives, eventually promoting the resolution of inflammation. Therefore, an improved understanding of the biochemistry of bioactive lipids at physiologic conditions will support the design of novel therapeutic strategies to promote resolution pathways (Barnig et al., 2019; Freire and Van Dyke, 2000; Gilroy and De Maeyer, 2015).

### 4.1 Limitations of the study

Due to the small number of donors, the statistical power of this study is limited, and many more functional correlations may be expected from more powerful studies. Furthermore, causal evidence for the described functional implications of bioactive lipids is lacking due to the inherent correlation-based approach, and require dedicated study designs for functional validation.

## 5 Conclusion

The aim of the study was to project functional knowledge established for proteins to co-occurring bioactive lipids such as fatty acids, endocannabinoids, or oxylipins, for which such knowledge is largely missing. The untargeted approach identified 168 bioactive lipids including a novel oxylipin, 4-HETE, which has now been listed by LIPID MAPS. The results of this correlation-based integration of proteomics and bioactive lipid analysis allowed us to validate the approach *via* the independent confirmation of previous knowledge. More importantly, numerous novel correlations were found suggesting the implication of various bioactive lipids in specific physiologic processes.

## Data Availability

The datasets presented in this study can be found in online repositories. The names of the repository/repositories and accession number(s) can be found in the article/Supplementary Material.
